# Corticoreticular Tract in the Human Brain: A Mini Review

**DOI:** 10.3389/fneur.2019.01188

**Published:** 2019-11-12

**Authors:** Sung Ho Jang, Sung Jun Lee

**Affiliations:** Department of Physical Medicine and Rehabilitation, College of Medicine, Yeungnam University, Daegu, South Korea

**Keywords:** corticoreticulospinal tract, corticoreticular tract, motor function, recovery mechanism, diffusion tensor tractography

## Abstract

Previous studies have suggested that the corticoreticular tract (CRT) has an important role in motor function almost next to the corticospinal tract (CST) in the human brain. Herein, the CRT is reviewed with regard to its anatomy, function, and recovery mechanisms after injury, with particular focus on previous diffusion tensor tractography-based studies. The CRT originates from several cortical areas but mainly from the premotor cortex. It descends through the subcortical white matter anteromedially to the CST with a 6- to 12-mm separation in the anteroposterior direction, then passing through the mesencephalic tegmentum and the pontine and pontomedullary reticular formations. Regarding its motor functions, the CRT appears to be mainly involved in the motor function of proximal joint muscles accounting for ~30–40% of the motor function of these joint muscles. In addition, the CRT is involved in gait function and postural stability. However, further studies that clearly rule out the effects of other motor function-related neural tracts are necessary to clarify the precise portion of the total motor function for which the CRT is responsible. With regard to recovery mechanisms for an injured CRT, three recovery mechanisms were suggested in five previous studies: recovery through the original pathway, recovery through perilesional reorganization, and recovery through the transcallosal pathway. However, each of those studies was single-case reports; therefore, further original studies including a larger number of patients are warranted.

## Introduction

The corticoreticulospinal tract is composed of the corticoreticular tract (pathway) (CRT) and the reticulospinal tract. The CRT is reported to originate mainly from the premotor cortex (PMC) and to terminate at the pontomedullary reticular formation (1–3). It innervates axial muscles and the proximal muscles of the extremities; therefore, it is involved in gait function and postural control (1, 2, 4).

In the human brain, the motor system can be categorized into pyramidal and extrapyramidal systems (2, 5). The extrapyramidal system includes the rubrospinal, reticulospinal, vestibulospinal, and tectospinal tracts (1, 2, 5). Although it has not been clearly elucidated, previous studies have demonstrated that the CRT is important in motor function, next to the corticospinal tract (CST), in the human brain (5–8). Therefore, detailed knowledge about the CRT is necessary for clinical neuroscience.

Functional neuroimaging and transcranial magnetic stimulation (TMS) techniques have been the main assessment tools used to investigate the CRT's functions (9–17). Using functional near-infrared spectroscopy (fNIRS), several studies have demonstrated that the PMC, which is the major site of origin of the CRT, is mainly activated during movements of the proximal joint muscles in normal subjects (9–11). Other studies using fNIRS have demonstrated that the enhanced PMC activation in the affected hemisphere is associated with locomotor recovery in stroke patients (12, 13). However, these studies had limitations such as that the selective movement of the proximal joint muscles is impossible during fNIRS and the CRT originated from other cortical areas such as the primary motor and prefrontal cortices as well as the PMC (18). Regarding TMS studies, many studies have suggested that the identity of the ipsilateral motor pathway from the affected cerebral cortex to the affected limbs that contribute to the motor recovery in the mature brain injuries such as adult stroke is most likely the CRT, and the characteristics of the motor-evoked potential of the ipsilateral motor pathway show delayed latency and smaller amplitude compared to those of the CST (14–16). However, there has been controversies on the identity and characteristics of motor-evoked potential of the ipsilateral motor pathway (15, 17). As a result, the above evaluation tools are limited as they do not allow visualization of the CRT within the human brain.

By contrast, diffusion tensor tractography (DTT), which is a derivative of diffusion tensor imaging (DTI), allows for visual reconstruction and estimation of the CRT three dimensionally in the human brain (3). The advantages of DTT compared to DTI is that the characteristics of the entire CRT can be evaluated by examining DTT parameters, in particular, the fractional anisotropy (FA), mean diffusivity (MD), and tract volume (TV) parameters (19, 20). In addition, changes in the status of the CRT can be estimated by performing DTT-based configurational analysis (21–24). As a result, DTT appears to be effective for research into CRT anatomy, function, and recovery mechanisms after injury in *in vivo* human brain. Thus far, many studies have used DTT to observe and report on those main research topics (9, 18, 19, 21–25).

The aim of this mini-review is to discuss the anatomy, function, and recovery mechanisms after injury of the CRT, with particular focus on previous studies using DTT to assess the CRT.

## Anatomy of the CRT

### Cortical Distribution of the CRT Origin

**Figure 1:**
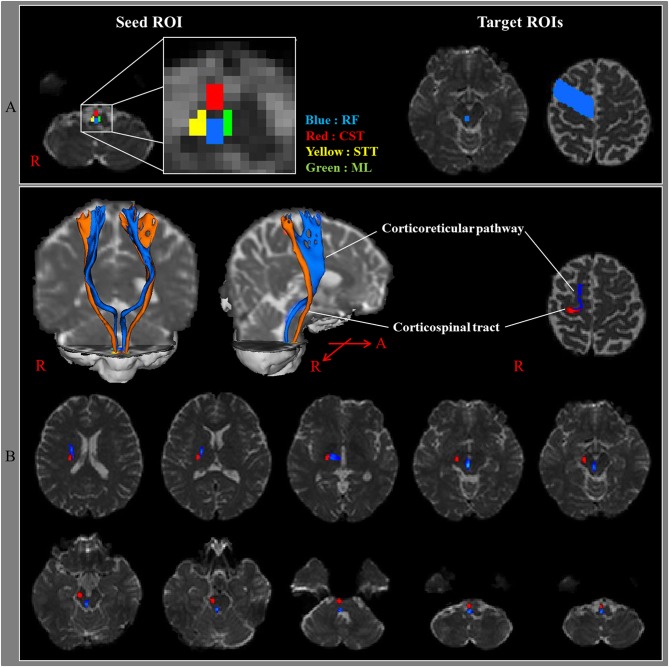
**(A)** Seed regions of interest (ROI) were given on the medullary reticular formation (blue rectangle). The first target ROI was given on the midbrain tegmentum (blue rectangle) and the second target ROI on the premotor cortex (Brodmann's area 6) (blue area). RF, reticular formation; CST, corticospinal tract; STT, spinothalamic tract; ML, medial lemniscus. **(B)** The pathways of the corticoreticular pathway (blue color) and CST (red color) are shown at each level of the brain in a normal subject (32-year-old male) [Reprinted with permission from Yeo et al. (3)].

Several animal studies have been conducted into the identification of the CRT of the cat brain (1, 4, 26). However, these studies did not provide detailed anatomical information on the CRT. However, there is a recent study that used DTT to reconstruct the CRT three dimensionally in 24 normal human subjects. In that study, the seed region of interest (ROI) was the medullary reticular formation while the first and second target ROIs were the midbrain tegmentum and the PMC, respectively (3). The results of this study revealed that the DTT-reconstructed CRT originated from the PMC, passed through the corona radiata and the posterior limb of the internal capsule (PL), to the mesencephalic tegmentum, the pontine reticular formation, and the pontomedullary reticular formation (Figure 1). The medial reticulospinal tract, which originated from the pontine nuclei of the reticular formation, descends in the medial aspect of the ventral funiculus of the spinal cord, whereas the dorsal (lateral) reticulospinal tract, arising from the medullary portion of the reticular formation, travels in the lateral funiculus of the spinal cord (2). Because the CRT that is reconstructed on DTT is terminated in the pontomedullary reticular formation, this CRT on DTT appears to be continued to the medial and lateral reticulospinal tracts (3). Excitability of spinal stretch reflex is maintained by a balanced control from inhibition by the dorsal reticulospinal tract and excitation by the medial reticulospinal tract and vestibulospinal tract (27, 28). The dorsal reticulospinal tract acts as a suprabulbar inhibitory system by receiving facilitation from the motor cortex *via* the CRT (27, 28). Hyperexcitability of the reticulospinal tract has been reported to be related to spasticity, abnormal motor synergy, and disordered motor control in stroke patients (27, 28).

The CST originates mainly from the primary motor cortex, whereas the CRT is reported to originate mainly from the PMC (1, 3, 6). Previous clinical studies have demonstrated that the PMC is the main cortical origin area for the CRT based on evidence that patients with PMC lesions show gait dysfunction or proximal muscle weakness (12, 13, 29–35). In contrast, other studies have demonstrated that the CRT originates from other cortical areas including the primary motor cortex, the prefrontal cortex, and the primary somatosensory cortex (9, 12, 13, 29, 36). Thus, the cortical distribution of the areas of origin of the CRT has not been clearly elucidated by these previous clinical functional neuroimaging studies; furthermore, there is only one study that used DTT to investigate this topic (18). In 2014, Jang and Seo (18), by applying DTT, reported on the cortical distribution of the areas of origin CRT in 42 normal subjects. In that study, CRTs originating from the primary motor cortex and PMC areas were reconstructed in both hemispheres (100%) of all subjects. However, CRTs originating from the primary somatosensory cortex and the prefrontal cortex were only reconstructed in 64 (76.2%) and 63 (75%), respectively, of the 84 hemispheres. The average TV values, which indicates the total number of fibers within a tract, for the CRT cortical origin areas were, in descending order, the PMC (1,177.3), the primary motor cortex (994.9), the primary somatosensory cortex (580.8), and the prefrontal cortex (575.7) (18, 20). The TV of the CRT from the PMC was significantly higher than those from the other cortical areas, and the TV of the primary motor cortex was significantly higher than those of the primary somatosensory cortex and prefrontal cortex areas. However, there was no significant difference between the TV values of the primary somatosensory cortex and prefrontal cortex areas.

### Anatomical Locations of the CRT at the Subcortical Level

**Figure 2:**
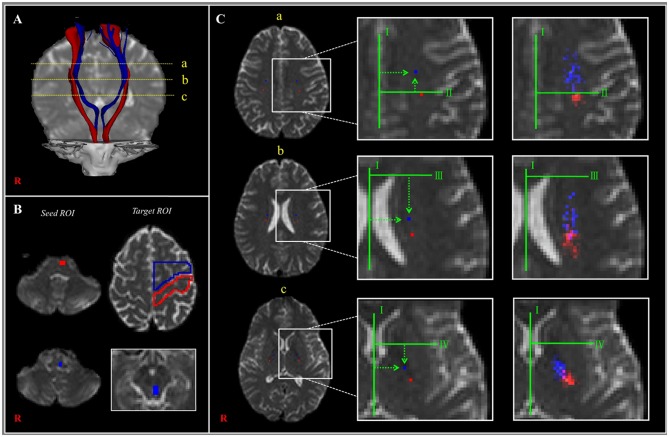
**(A)** The corticoreticular pathway (CRP) (blue color) and corticospinal tract (CST) (red color) were reconstructed in both hemispheres [a—centrum semiovale (CS) level, b—corona radiata (CR) level, c—posterior limb of the internal capsule (PL) level]. **(B)** The regions of interest (ROIs) (blue color) were set for the CRP (seed—reticular formation of the medulla, first target ROI—midbrain tegmentum, second target ROI—Brodmann's area 6). The ROIs (red color) were set for the CST (seed ROI—anterior portion of lower pons, target ROI—Brodmann's area 4). **(C)** Landmarks used in determining the location of the CRP and CST (left and middle columns). The probabilistic maps for the CRP and CST were superimposed on images of a subject (right column). Line I: the midline between the right and left hemispheres for measurement of mediolateral distance. Line II: the horizontal line from the most medial point of the central sulcus to the midline as the reference line for the anteroposterior direction. Line III: the horizontal line that passes through the anterior end of the lateral ventricle for the reference line for the anteroposterior direction. Line IV: the horizontal line from the middle point at the genu of the internal capsule [Reprinted with permission from Jang and Seo (25)].

Little has been reported about the anatomical location of the CRT as it passes along its descending pathway (25). To the best of our knowledge, only one study, which was reported in 2015 by Jang and Seo (25), has described the anatomical locations of the CRT in the centrum semiovale, corona radiata, and PL areas, and the authors used DTT observations to compare those locations with the CST locations in 33 normal subjects. The detailed anatomical locations of the CRT, based on that study, are described in Table 1 and presented in Figure 2. Compared to the CST, the CRT is located anteromedially in all three subcortical white matter regions, and the mean distances between the CRT and CST ranged between 6 and 12 mm in the anteroposterior direction but with overlapping of some portions of the CRT and CST (Figure 2; Table 1).

**Table 1:** Average distances of the highest probability points of the corticoreticular pathway and corticospinal tract.

	**Corticoreticular pathway**		**Corticospinal tract**	
	**Anterior to posterior distance (mm)**	**Medial to lateral distance (mm)**	**Anterior to posterior distance (mm)**	**Medial to lateral distance (mm)**
CS	13.34 (±5.84)	20.55 (±2.79)	−0.60 (±2.26)	22.88 (±2.05)
CR	−21.94 (±7.04)	22.57 (±2.22)	−33.87 (±4.87)	23.75 (±2.05)
PL	−15.65 (±3.12)	16.38 (±1.90)	−21.78 (±2.21)	21.70 (±2.04)

*Values indicate mean (±standard deviation); CS, centrum semiovale; CR, corona radiata; PL, posterior limb of the internal capsule [Reprinted with permission from Jang and Seo (25)]*.

## Functions of the CRT

The lateral CST is mainly involved in the control of the movements of distal extremities, whereas the CRT innervates axial muscles and the proximal muscles of the extremities; as a result, the CRT is involved in gait function and postural stability (1, 2, 4, 6–8, 37–41).

### Motor Function

That the CRT is mainly involved in the motor functions of proximal joint muscles was demonstrated by Jang et al. (39) in 2015 by using DTT. The authors investigated the relationship between motor weakness and DTT parameters in 17 patients who showed intact CSTs with full tract integrity following subarachnoid hemorrhages (39). They found that the FA value, which indicates the degree of directionality and integrity of white matter microstructures, of the CRT was correlated with the motor functioning of the shoulder and hip, but they did not detect a correlation between the FA value and the motor functioning of the distal joint muscles (i.e., elbow, hand, knee, and ankle) (20, 39). As a result, the authors concluded that the CRT injury severity in these patients was correlated with motor weakness in their proximal joint muscles (shoulder and hip).

Several studies have reported on the characteristics of motor weakness due to CRT injury in patients with brain injury (7, 8, 40, 41). However, precise estimation of the portion of the affected motor function for which the CRT is responsible is difficult because patients with brain injury usually have concomitant simultaneous injuries of other neural tracts associated with motor function such as the CST or the corticofugal tracts from the secondary motor area (6, 42). However, among the previous studies that have reported on CRT injury, to the best of our knowledge, only two studies have demonstrated that the CST, which is the most significant tract for motor function, was spared from brain injury (6, 7). In 2011, Chen et al. reported on a patient who revealed proximal quadriparesis [right/left proximal muscle power, Medical Research Council (MRC) scale scores: 4^−^/3] due to a right lateral medullary infarction, but on conventional brain MRI, they observed that the CST was spared in both anterior medullas (43). Subsequently, Do et al. (7) reported on a patient with a cerebral infarct in the left frontal lobe that involved the PMC who exhibited an intact CST in both hemispheres—results that were based on their DTT assessment of tract integrity and the motor-evoked potential parameters obtained for the patient's hand muscles. The patient exhibited greater weakness in the right proximal joint muscles (MRC: shoulder abductor, 4^−^; hip flexor, 4^−^) than in the right distal joint muscles (4^+^) (44), and, on DTT, discontinuation of the left CRT was detected at the PL.

However, other studies could not describe the status of the CST clearly (8, 40, 41). In 2013, Yeo et al. reported on a patient who showed proximal weakness (MRC scores: shoulder abductor, 3^+^; hip flexor, 3^+^) following traumatic contusional hemorrhage (41, 43). Subsequently, Yoo et al. reported that the total motor function of 13 patients with CRT injury but preserved integrity of the CST following putaminal hemorrhage averaged 66.3 ± 7.3 on the Motricity Index (MI; maximum score: 100) (40, 44). During the same year, Kwon and Jang reported on a patient who showed proximal weakness following traumatic brain injury (MRC scores: shoulder abductor: 3^+^/3^+^, and hip flexor: 3/3^+^) (43, 45). In 2015, Lee and Jang reported that 29 patients with proximal weakness following mild traumatic brain injury exhibited more severe weakness in the proximal muscles (average MRC score: shoulder abductor: 3.66, hip flexor: 3.51) than in the distal muscles (MRC scores: finger flexor: 4.03, finger extensor: 4.11, knee extensor: 4.01, and ankle dorsiflexor: 4.07) (8, 43). Considering the results of the above studies into the motor functions of the CRT, it appears that the CRT is mainly involved in the motor functions of proximal joint muscles, accounting for ~30–40% of the total motor function of these joint muscles. However, further studies that can clearly rule out the effects on the CST and the corticofugal tracts from the secondary motor area are needed in order to clarify the precise portion of the total motor function for which the CRT is responsible (6, 42).

### Gait Function

Following the introduction of DTT, several studies have used DTT to demonstrate that gait function in patients with brain injury is closely related to changes in the CRT in the affected hemisphere (19, 46–48). In 2013, Jang et al. (19) investigated the relationship between the CRT and gait ability in chronic hemiparetic stroke patients. The authors classified the patients, who had been confirmed on DTT to have complete injury of the CST (discontinuation of the CST around or below the lesion) in the affected hemisphere, into two groups based on their ability to walk (one group: patients who could walk; the other group: patients who could not walk). No difference was detected in the DTT parameters of the CRT in the affected hemisphere between the two patient groups; however, in the unaffected hemisphere, patients who could walk had higher TV values (an indicator of the total fiber number of the CRT) than those in patients who could not walk as well as a normal control subject. In addition, the TV value for the CRT in the unaffected hemisphere was correlated with gait ability (19). In contrast, the DTT parameters for the CST in the unaffected hemisphere showed no correlation with gait ability. Based on their results, the authors concluded that activation of the CRT in the unaffected hemisphere appears to be a mechanism for gait recovery after stroke (19). Recently, two patients showed gait recovery upon activation of the CRT in the unaffected hemisphere following intracerebral hemorrhage (46, 47). By contrast, degeneration of both CRTs has been demonstrated in a patient who presented with delayed gait deterioration following spontaneous putaminal hemorrhages in both hemispheres (48).

### Postural Stability

In 2016, Jang et al. (38) reported on an investigation into postural instability in 25 mild traumatic brain injury patients who were demonstrated on DTT to have CRT injuries. The authors examined postural instability in terms of the patients' Balance Error Scoring System scores, the displacement of their centers of pressure, as well as the TV values of their CRTs and observed that both the patients' postural stability and TV values were decreased in these patients.

## Recovery Mechanisms of Injured CRT

Three different recovery mechanisms of injured CRT in patients with brain injury have been suggested in several studies using DTT of the CRT (21–24, 49, 50). These mechanisms include the recovery of a CRT injury along the original pathway, recovery through perilesional reorganization, and recovery along the transcallosal pathway (21–24, 49, 50). However, this list may be incomplete as these studies were all single-case reports. In addition, these studies except for one study did not describe the CST state, which is a major neural tract for motor function (21–24, 49, 50).

### Recovery of CRT Injury Along the Original Pathway

Regarding the recovery of an injured CRT along its original pathway, only two cases describing this mechanism have been reported (21, 24). In 2013, Yeo and Jang (24) reported on a patient who showed recovery of an injured CST and an injured CRT following intracerebral hemorrhage in the left subcortical white matter; tract recovery was demonstrated on follow-up DTTs. The patient showed a thin left CST and a discontinued left CRT at the midbrain level at 3-weeks post-onset; thickening of the left CST and restoration of the integrity of the left CRT were observed at 6-weeks post-onset, along with concurrent motor recovery. Subsequently, Jang and Lee (21) reported on a patient who showed recovery of injured CRTs after traumatic subdural hematoma in the right frontoparietal lobes. DTT at 10-weeks post-onset revealed that both CRTs of the patient were discontinuous at the subcortical white matter (centrum semiovale), with only a few CRT fibers appearing to be connected to the cerebral cortex in the left hemisphere. By contrast, on 20-week post-onset DTT, the discontinued right CRT was seen to have elongated to the cerebral cortex, and the previously narrow portion of the left CRT had thickened. However, the authors did not describe the CST state. Recently, Jang et al. (50) demonstrated recovery of injured CRTs along with recovery of leg weakness in a patient with spontaneous subarachnoid hemorrhage and intraventricular hemorrhage without description on the CST state. After onset, the patient presented with complete weakness of both legs (MRC score: 0/0). That weakness underwent slow recovery until 11-months after onset when the patient was able to walk on stairs (MRC score: 4/4). The discontinuations observed in both CRTs on 1-month DTT were less obvious on 3-month DTT with connections to the cerebral cortex being observed. Further tract thickening was observed on 6- and 20-month post-onset DTT.

### Recovery of CRT Injury *via* Perilesional Reorganization

In 2015, Jang et al. (22) reported on a patient with a right CRT injury due to a right middle cerebral artery territory infarction. Although the patient showed complete paralysis of the left upper and lower extremities (MRC score: 0), the weakness of his left leg showed good recovery at 10-weeks after onset (MRC scores: hip flexor, 4^−^; knee extensor, 4^−^). On 4-week post-onset DTT, the right CRT was discontinuous at the corona radiata level. However, on 10-week DTT, the discontinued right CRT was shown to have its integrity restored to the PMC through the lateral ventricular wall, an abnormal anatomical location for a CRT. As a result, the authors concluded that the injured CRT appeared to have recovered through peri-infarct reorganization. However, the authors did not describe changes of the CST.

### Recovery of CRT Injury *via* the Transcallosal Pathway

**Figure 3:**
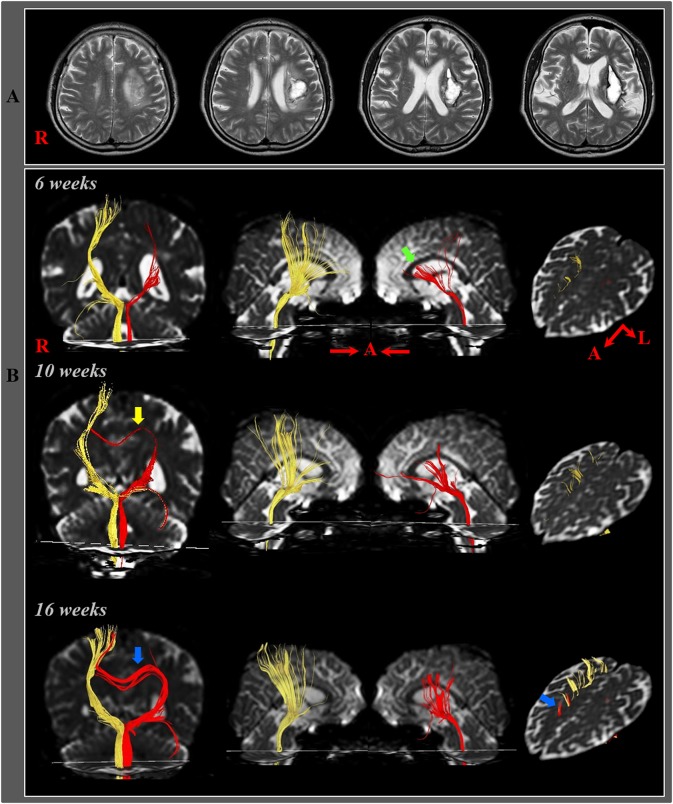
**(A)** T2-weighted MR images showed a hematoma in the left corona radiata and basal ganglia at 6-weeks after onset. **(B)** Results of diffusion tensor tractography (DTT). On 6-, 10-, and 16-week DTTs, the CRPs in the right hemispheres originated from the premotor cortex and primary motor cortex and descended through the known CRP pathway. By contrast, in the left (affected) hemisphere, the CRP showed severe narrowing with discontinuation of the anterior fibers (green arrow) on 6-week DTT, however, the discontinued fibers of the CRP were connected to the corpus callosum through the transcallosal fibers (yellow arrow) on 10-week DTT and became thicker and connected to the right cerebral cortex *via* the corpus callosum (blue arrow) on 16-week DTT [Reprinted with permission from Jang and Yeo (23)].

In 2014, Jang and Yeo (23) demonstrated recovery of an injured CRP along the transcallosal pathway in a patient with a putaminal hemorrhage. The patient presented with complete paralysis of the left extremities (MRC score: 0). At 16-weeks after onset, his right hemiplegia had recovered well, but there was severe proximal weakness (MRC scores: shoulder abductor; 3, finger extensor; 4, hip flexor; 3^+^, ankle dorsiflexor; 4). On 6-week DTT, the left CRT was shown to have discontinuous anterior fibers at the corona radiata. However, on 16-week DTT, the previously discontinuous anterior CRT fibers were shown to be connected to the right cerebral cortex *via* transcallosal fibers (Figure 3). As a result, the authors concluded that injury recovery *via* the transcallosal pathway might be a recovery mechanism for an injured CRT. However, the authors did not describe the CST state.

In 2016, Jang and Chang (49) reported on a patient who showed recovery of a CRT injury *via* transcallosal fibers following spontaneous intracerebral hemorrhage in the bilateral pontine tegmentum. The patient could not walk at 2-weeks after onset but was able to walk independently with good recovery of motor weakness of both legs at 6-weeks post-onset. On 2-week post-onset DTT, the right CRT was discontinuous at the midbrain level, and the left CRT could not be reconstructed by DTT. However, on 6-week DTT, the right CRT was shown to be connected to the right PMC and with tract thickening. Furthermore, transcallosal fibers originating from the right CRT and descending below the corpus callosum in the left hemisphere were observed. The authors suggested that the right CRT appeared to have undergone recovery *via* its original pathway as well as along the transcallosal pathway. However, the authors did not consider changes of the CST.

## Conclusions

This article reviewed the CRT in terms of its anatomy and function and the recovery mechanisms after CRT injury, with particular focus on previous studies that have used DTT to examine the CRT. The CRT originates mainly from the PMC but the tract also has origins in other cortical areas (the M1, S1, and prefrontal cortex areas) (9, 12, 13, 18, 29, 36). From the tract's origin areas, it passes through the corona radiata and the PL anterior to the CST. The CRT is located anteromedially to the CST, typically within approximately 6–12 mm in the anteroposterior direction (25). The CRT then passes through the mesencephalic tegmentum, the pontine reticular formation, and the pontomedullary reticular formation (3). Based on the results of previous studies into CRT injuries, the CRT appears to be mainly involved in the motor functions of proximal joint muscles, accounting for ~30–40% of motor function in these joint muscles (7, 8, 39–41). In addition, the CRT is involved in gait function and postural stability (19, 38, 46–48). However, further studies that clearly rule out the motor function effects of other neural tracts are needed to clarify accurately the portion of the total motor function for which the CRT is responsible (6, 42). With regard to the potential recovery mechanisms for injured CRTs, thus far, three recovery mechanisms have been suggested in previous DTT studies: recovery of the injured CRT along its original pathway, recovery *via* perilesional reorganization, and recovery along the transcallosal pathway (21–24, 49, 50). However, these studies are all case reports on a total of six patients; therefore, further original studies involving a larger number of patients are warranted. In addition, further studies that combine DTT results with those from transcranial magnetic stimulation and functional neuroimaging techniques are needed in order to clarify further the CRT motor functions within the normal brain and further elucidate the full range of recovery mechanisms of the CRT following brain injury.

## Author Contributions

SJ: study concept and design, manuscript development, writing, and funding. SL: critical revision of manuscript for intellectual content.

### Conflict of Interest

The authors declare that the research was conducted in the absence of any commercial or financial relationships that could be construed as a potential conflict of interest.
